# Neck of Femur Fracture in a Patient with a Chronic Osteomyelitis of the Ipsilateral Foot

**DOI:** 10.1155/2016/2108537

**Published:** 2016-10-24

**Authors:** Anne-Carolin Döring, Anne J. H. Vochteloo, Kees van Doorn, Rianne M. H. A. Huis in 't Veld, Anil Peters

**Affiliations:** Centre for Orthopaedic Surgery, OCON, P.O. Box 546, 7550 AM Hengelo, Netherlands

## Abstract

This case report describes a successful two-stage treatment in a 75-year-old male with a displaced neck of femur fracture, also suffering from an active chronic osteomyelitis of the ipsilateral calcaneus. In our case, a below-knee amputation was performed first, followed by total hip arthroplasty two weeks later. At 15-month follow-up, full recovery of the prefracture level of activities of daily living without significant impairment was obtained. Only a few cases of total hip arthroplasty in amputees have been published, but the indication for surgery was mainly traumatic or advanced osteoarthritis. Treating patients with this type of comorbidities is challenging; therapeutic dilemmas can be major. The management in cases like these requires a thorough evaluation and a clear surgical and medical treatment plan, preferably conducted by a multidisciplinary orthogeriatric team.

## 1. Introduction

Hip fracture patients often suffer from comorbidities. We discuss the therapeutic dilemmas associated with surgical treatment of a hip fracture in patients with chronic, active infections: in this case an active, chronic osteomyelitis of the ipsilateral heel. The principles of clinical assessment are highlighted and a potential treatment algorithm is proposed.

## 2. Case Report

**Figure 1:**
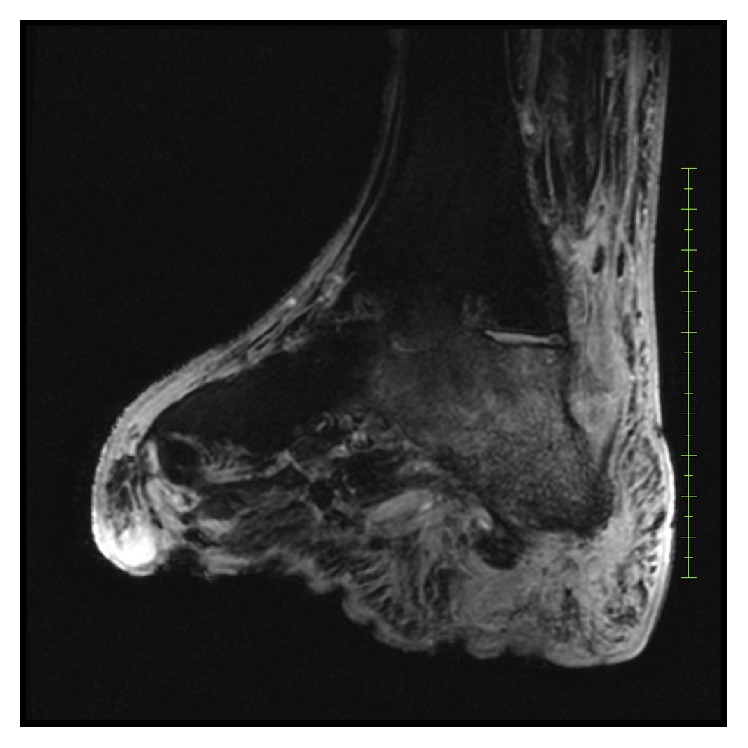
Magnetic Resonance Imaging demonstrating chronic osteomyelitis of the right calcaneus.

A 75-year-old Caucasian male presented to the Emergency Department (ED) after a fall, complaining of pain in his right hip and he was unable to bear weight. His most important comorbidities were severe peripheral vascular disease, treated with percutaneous transluminal angioplasty of the left common iliac artery, and a forefoot amputation of the right foot as a final treatment option for a chronic, progressive, and infected ulcer of the right hallux. A new ulcer had developed on the ipsilateral heel. Despite multiple courses of antibiotic treatment, the patient developed osteomyelitis of the right calcaneus. This was confirmed by Magnetic Resonance Imaging (MRI), which demonstrated osteomyelitic changes without a sequester (Figure 1). The ulcer was initially treated with a below-knee cast and oral Clindamycin, with good results. Follow-up at one year, however, revealed recurrence of the infection. A chronic osteomyelitis developed, despite long-term courses of antibiotic therapy and surgical debridement, combined with another period of cast immobilization.

**Figure 2:**
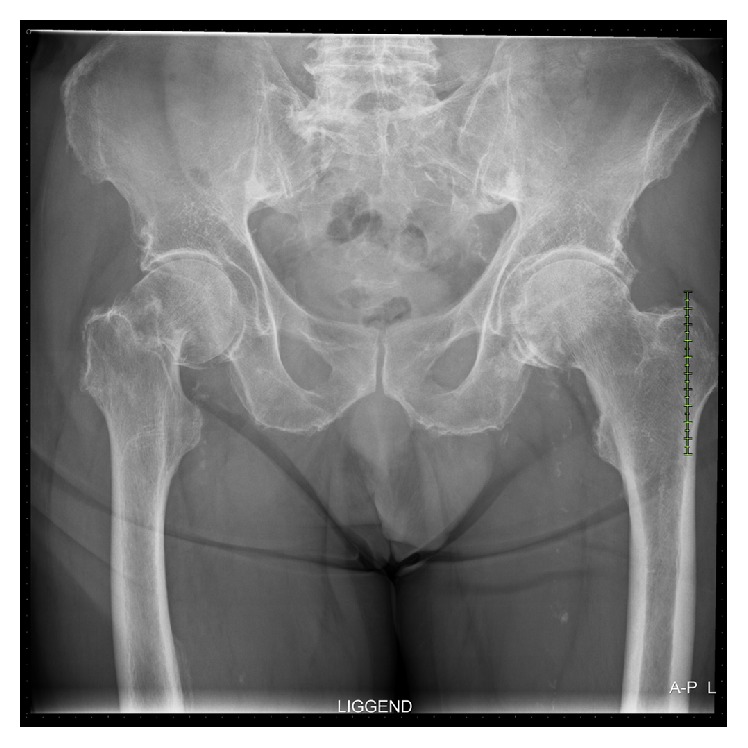
Anterior-posterior X-ray pelvis: displaced neck of femur fracture (right).

At examination on the ED, the right hip was painful and the leg was shortened and externally rotated. The ulcer on the right heel showed two cutaneous fistulas, both sanious but not infected. Radiographs demonstrated a displaced neck of femur fracture (Garden Type III; AO classification 31-B3) (Figure 2). Blood tests on admission revealed a white blood cell (WBC) count of 6.4 × 10^9^/L and a C-reactive protein (CRP) level of 15 mg/L.

A chronic ulcer with an active osteomyelitis in a patient with a hip fracture requiring surgery provided our team with a challenging therapeutic dilemma. Surgical treatment, whether closed reduction and internal fixation or (hemi)arthroplasty, is the gold standard for a displaced neck of femur fracture [1]. However, the risk of deep infection of the implant and sepsis is high in a patient with active, chronic osteomyelitis, especially in the ipsilateral extremity.

**Figure 3:**
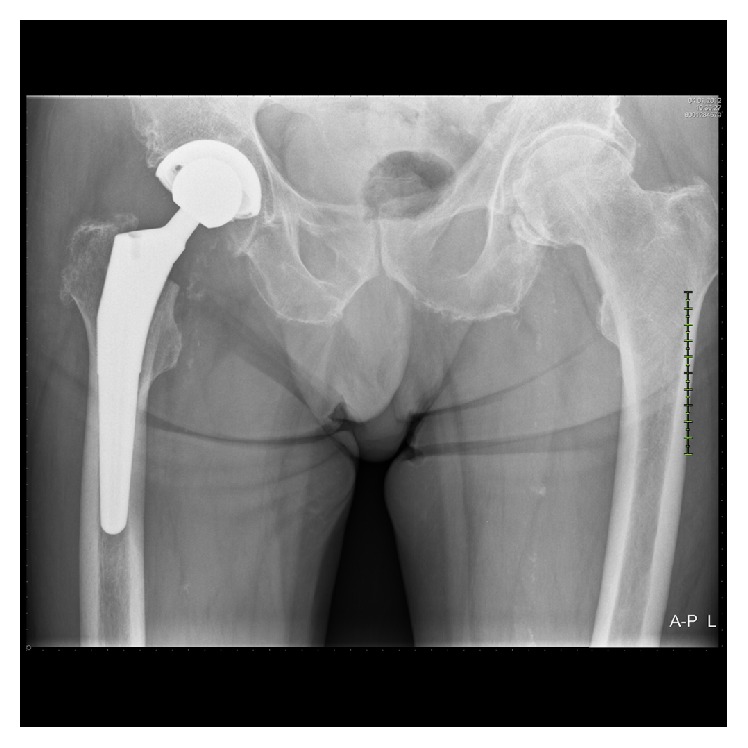
Uncemented total hip arthroplasty without signs of loosening at 15-month follow-up.

**Figure 4:**
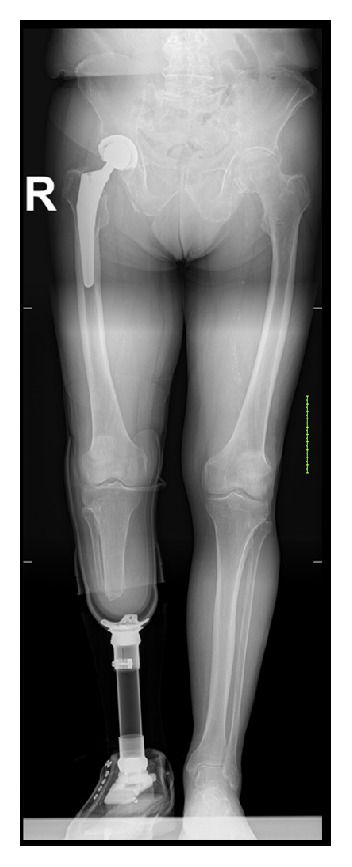
Uncemented total hip arthroplasty with the lower leg prosthesis.

Our multidisciplinary trauma team reached a consensus to eliminate the potential source of infection first by performing a below-knee amputation. The wound healed without any complications with a stump length of about 14 cm. Uncemented total hip arthroplasty (THA) with a 36-millimetre diameter head (Exceed ABT acetabular system and Taperloc hip system, Biomet, Warsaw, USA) was conducted two weeks later. The patient recovered well, following an intensive rehabilitation programme. At the follow-up at 15 months and 4 years after operation, he had returned to the prefracture level of activities of daily living without significant impairment. X-rays demonstrated no signs of loosening (Figures 3 and 4).

## 3. Discussion

Hip fractures in the elderly are challenging injuries to treat, especially with the presence of a chronic, active osteomyelitis as in our case. The therapeutic dilemmas and management decisions are interesting and clinically important.

The main question was whether to perform surgery to treat the hip fracture first (within 48 hours, as is common practice) or to start treatment of the active osteomyelitis, being a potential source of surgical site infection, initially. However, surgical and medical treatment of chronic osteomyelitis may follow a protracted course, delaying fracture treatment, which is associated with significant morbidity and mortality [2].

Treatment of a displaced femoral neck fracture can be either by internal fixation (IF) or by (hemi)arthroplasty. The advantage of IF in our case is that it can be performed immediately, combined with prolonged antibiotic therapy. In case of the occurrence of a deep infection of the hip, one could remove the metal components as soon as healing of the fracture is observed. Disadvantage of this treatment strategy is that, in case of an infection, short-term functional outcome is poor and mortality increases [3].

Although the most recent meta-analysis did not show a significant difference in outcome between IF and total hip arthroplasty (THA) in the treatment of hip fractures in the elderly, our national guideline recommends the use of THA in elderly with a displaced femoral neck fracture, as does the NICE guideline in the UK [1, 4, 5].

The current literature on THA in patients with an amputation of the lower extremity consists mainly of small case series in which posttraumatic or advanced osteoarthritis of the hip is the main indication for surgery [6–8]. Lower limb amputees may experience excessive joint reaction forces and demonstrate a relatively high incidence of severe osteoarthritis particularly in the ipsilateral hip and the contralateral side [9, 10]. One case report describes the relatively close scheduled surgeries of a below-knee amputation for osteomyelitis of the right heel, followed by ipsilateral THA for posttraumatic osteoarthritis secondary to an acetabular fracture [11].

Internal fixation of a displaced neck of femur fracture in patients with an ipsilateral below-knee amputation is difficult [12]. Due to altered biomechanics (i.e., increased proximal excursion of the hip, lateralization of the centre of gravity, and increased shear forces) at the fracture site in patients with below-knee amputations, a high risk of fixation failure or nonunion exists [6]. The use of (bipolar) hemiarthroplasty or THA is therewith recommended for this population. A case series of four patients with an amputated lower extremity who were treated with THA for osteoarthritis or a fracture demonstrated good clinical outcome after a well-guided rehabilitation programme. [11]. Part of this programme is enforcement of standard postoperative hip precautions to avoid dislocation of the THA during placing and removing of the prosthesis [11].

We did not consider a Girdlestone procedure as a viable option in our patient because of the poor functional outcome in the elderly [13].

To conclude, this case report presents the successful treatment of a patient with a displaced neck of femur fracture, also suffering from an active, chronic osteomyelitis of the ipsilateral calcaneus. A two-stage approach was chosen: initially our patient underwent a below-knee amputation, followed by a THA two weeks later. The final treatment choice should always depend on the characteristics of the patient and the degree of the infection.
